# Whole Genome Sequence Dataset of Mycobacterium tuberculosis Strains from Patients of Campania Region

**DOI:** 10.1038/s41597-024-03032-6

**Published:** 2024-02-19

**Authors:** Veronica Folliero, Carlo Ferravante, Valentina Iovane, Annamaria Salvati, Laura Crescenzo, Rossella Perna, Giusy Corvino, Maria T. Della Rocca, Vittorio Panetta, Alessandro Tranfa, Giuseppe Greco, Teresa Baldoni, Ugo Pagnini, Emiliana Finamore, Giorgio Giurato, Giovanni Nassa, Mariagrazia Coppola, Luigi Atripaldi, Rita Greco, Annamaria D’Argenio, Maria Grazia Foti, Rosamaria Abate, Annalisa Del Giudice, Bruno Sarnelli, Alessandro Weisz, Giuseppe Iovane, Renato Pinto, Gianluigi Franci, Massimiliano Galdiero

**Affiliations:** 1 Department of Veterinary Medicine and Animal Productions, University of Naples Federico II, Naples, Italy; 2 Department of Experimental Medicine, University of Campania “Luigi Vanvitelli”, Naples, Italy; 3 Department of Medicine, Surgery and Dentistry “Scuola Medica Salernitana”, Baronissi, SA Italy; 4 Molecular Pathology and Medical Genomics Program, San Giovanni di Dio e Ruggi D’Aragona University Hospital, Salerno, Italy; 5 Department of Agricultural Sciences, University of Naples Federico II, Portici, Naples, Italy; 6 Laboratory of Microbiology and Virology, Ospedali dei Colli, Naples, Italy; 7 UOC Microbiology - Ospedale Cardinale Ascalesi, ASL NA1 Naples, Italy; 8 UOSD Microbiology - AORN Sant ‘Anna and San Sebastiano, Caserta, Italy; 9 UOC Microbiology and Virology- San Giuseppe Moscati Hospital, Avellino, Italy; 10 Clinical Pathology and Microbiology Unit, San Giovanni di Dio e Ruggi D’Aragona University Hospital, Salerno, Italy; 11 UOC Virology and Microbiology – University Hospital AOU “Luigi Vanvitelli”, Naples, Italy; 12 UOD Prevenzione e Sanità Pubblica Veterinaria, Direzione Generale Tutela della Salute – Regione Campania, Naples, Italy

**Keywords:** Tuberculosis, Diagnosis

## Abstract

Tuberculosis (TB) is one of the deadliest infectious disorders in the world. To effectively TB manage, an essential step is to gain insight into the lineage of *Mycobacterium tuberculosis* (MTB) and the distribution of drug resistance. Although the Campania region is declared a cluster area for the infection, to contribute to the effort to understand TB evolution and transmission, still poorly known, we have generated a dataset of 159 genomes of MTB strains, from Campania region collected during 2018–2021, obtained from the analysis of whole genome sequence. The results show that the most frequent MTB lineage is the 4 according for 129 strains (81.11%). Regarding drug resistance, 139 strains (87.4%) were classified as multi susceptible, while the remaining 20 (12.58%) showed drug resistance. Among the drug-resistance strains, 8 were isoniazid-resistant MTB, 4 multidrug-resistant MTB, while only one was classified as pre-extensively drug-resistant MTB. This dataset expands the existing available knowledge on drug resistance and evolution of MTB, contributing to further TB-related genomics studies to improve the management of this disease.

## Background & Summary

**Fig. 1:**
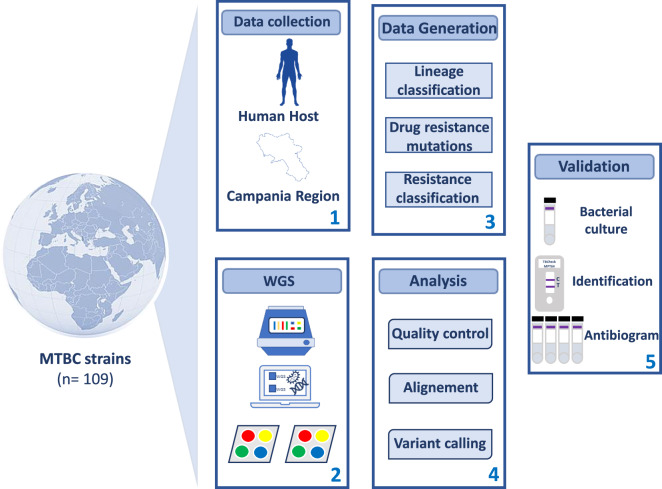
Study workflow. Data collection and procedure pipeline are shown.

**Fig. 2:**
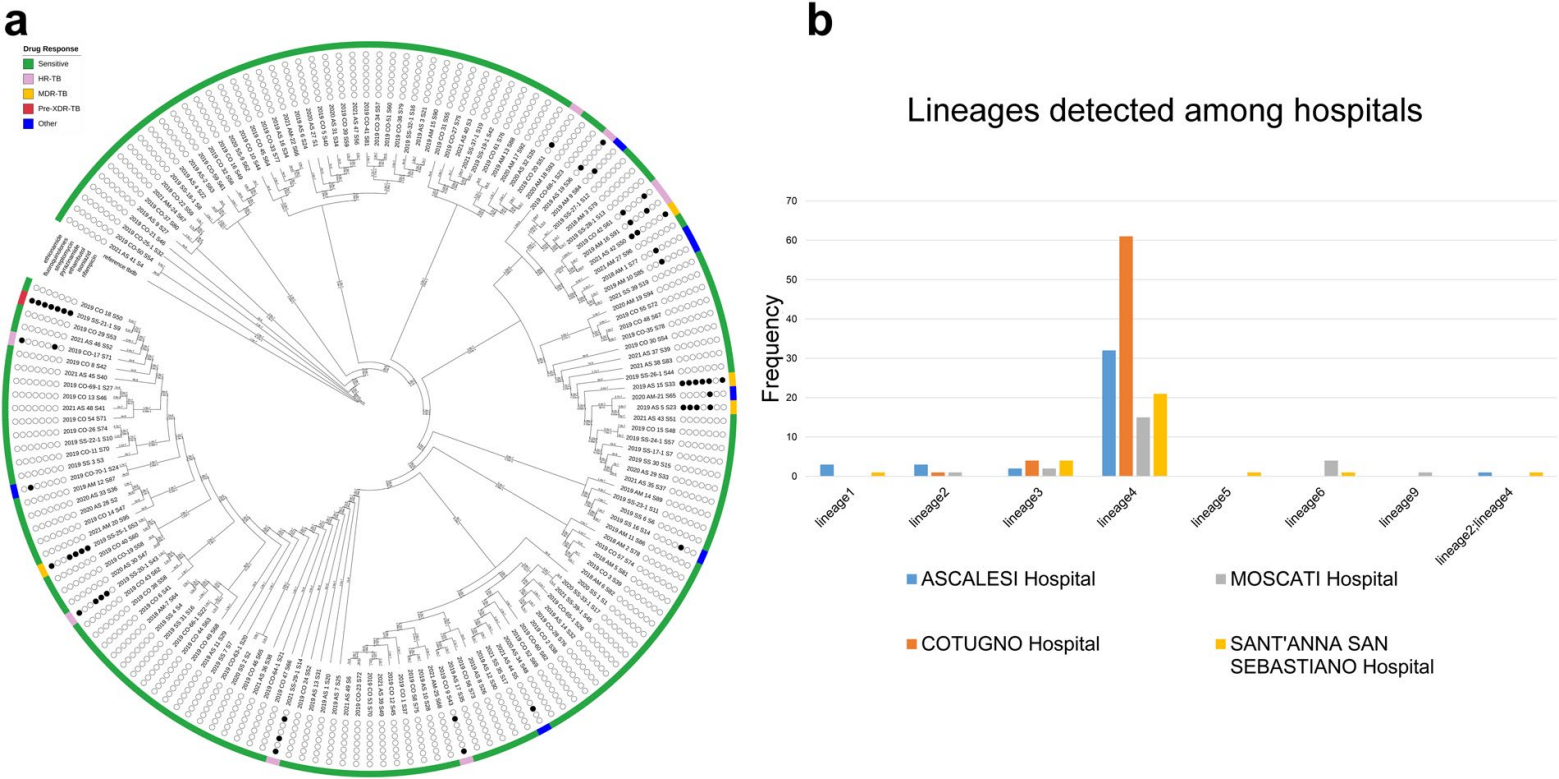
*In-silico* profiling of MTB isolates with lineages assignment and drug-resistance analysis results. Circular tree reporting the *in-silico* prediction of the resistance to the tested antibiotics and the phylogenetic distance that characterized the 159 MTB isolates. The 159 MTB isolates were classified as sensitive (green) (n = 139), HR-TB (light purple) (n = 8), Other (blue) (n = 7), MDR-TB (orange) (n = 4) and Pre-XDR-TB (red) (n = 1) (**a**). Histogram plot showing the distribution of all lineages among the four hospitals enrolled in this study (**b**).

Tuberculosis (TB) is a major global health threat that affects millions of people worldwide and has significant social and economic impacts^1^. In 2021, an estimated 10.6 million people were affected by TB, and 1.6 million deaths were reported globally (https://www.who.int/news-room/fact-sheets/detail/tuberculosis). The direct healthcare costs of TB are also high, with an average of $567,708 per TB case^2^. In Italy alone, 2146 cases were reported^3^. TB is caused by *Mycobacterium tuberculosis* complex (MTBC)^4^, comprising different lineages with varying geographical locations and spreads^5^. MTBC includes *Mycobacterium tuberculosis* (MTB) *sensu stricto*, which includes lineages 1, 2, 3, 4, 7, and 8, and MTB var. africanum, comprising lineages 5, 6, and 9^6^. Lineage 1 is widespread in East Africa, while lineage 2 is highly mobile, spreading in Asia, Africa, and Europe^7^. Lineage 3 is mainly located in southern Asia and northern and eastern Africa, while lineage 4 is common in Europe and southern Africa^8^. Lineages 5, 6, and 7 are endemic in West Africa and Ethiopia^9^. In recent years, new lineages 8 and 9 have been detected in central and east Africa, respectively^10^. Several evidences report that lineages differ in transmission, progression and severity of the disease caused, vaccine, diagnosis and drug efficacy, and drug resistance^11^. Indeed, lineages 5 and 6 are closely associated with extrapulmonary infections, while variant 4 is more related to pulmonary manifestations^12^. Different studies have highlighted the immunological recovery of patients infected with lineage 6 MTB, compared with those with lineage 4 MTB^13^. In contrast to the other lineages, lineage 6 responds more slowly to treatment with first-line drugs^14^. The latter grows more slowly *in vitro* and is more associated with a false negative culture^9^. Lineages 3, 4, and 5 have more virulence factors than lineage 7^15^. Moreover, lineages 2 and 3 have a strong propensity to acquire gene determinants of drug resistance^16^. Multidrug-resistant tuberculosis (MDR-TB) poses a serious threat to public health. In 2021, approximately 450,000 MDR-TB cases occurred, resulting in 191,000 deaths worldwide. Standard first-line treatment is hardly ineffective for MDR-TB patients. Indeed, only about 1 in 3 MDR-TB patients had access to appropriate treatment in 2021^17^. Monitoring the spread of different lineages and drug-resistant strains is crucial to improve TB control. The GENEXPERT MTB/RIF test is broadly exploited in most hospitals in the country. This assay fails to discriminate lineages and highlights only rifampicin resistance^18^. Whole genome sequencing (WGS) has become an essential tool to acquire comprehensive genetic information regarding strains of TB, leading to improved disease control and containment of its global health impact^19^. The characterization of genetic diversity in locally detected MTBC strains through WGS is crucial for understanding the transmission and evolution of TB drug resistance in Italy. In our study, we aimed to provide a comprehensive dataset of MTBC-positive individuals, which would enable further investigation of the impact of MTBC infection on the population. To achieve this, we sequenced and analysed the genomes of 159 MTB isolates, obtained from patients in the Campania region during 2018–2022. Our analysis focused on genetic diversity and on the identification of variants associated with drug resistance. The study design and data collection process are illustrated in Fig. 1. Notably, through WGS analysis, we successfully identified drug response and resistance (Fig. 2a) as well as several lineages spread across the four participating hospitals (Fig. 2b). This approach also allowed us to observe the distribution of region-specific MTB variants, which can contribute to infection monitoring efforts. The proposed WGS dataset provides valuable insights into the biological impact of MTB distribution. Researchers can analyse this dataset in conjunction with others to identify key lineages and significant gene mutations associated with drug resistance. Furthermore, the dataset includes various clinical factors (such as patients’ provenance, starting biological material, and antibiogram assay results) that can be utilized to investigate the relationship between these factors and MTB infection. The identification of a diverse and heterogeneous population of MTB lineages, along with the presence of antibiotic resistance, offers to the researchers a wealth of data to conduct versatile studies. These findings can be instantly accessed to facilitate correlation studies between phenotypic and genotypic data, enabling the identification of drug-resistance mutations and markers associated with disease progression. Overall, our data could implement available studies more effectively, improving TB management.

## Methods

### Sample cohort

This study involved 159 MTBC isolates collected retrospectively between 2018 and 2021 from four hospitals in the Campania region (Southern Italy). In detail, 41 strains came from Ascalesi hospital of Naples (Via Egiziaca A. Forcella, 31–80144 Napoli (NA)), 66 isolates from Cotugno hospital (Via Quagliariello, 54–80131 Napoli (NA). Twenty-three strains were enrolled at AORN S.G. Moscati (Contrada Amoretta - 83100 Avellino (AV)), and 29 isolates were collected at AO Sant’Anna e San Sebastiano of Caserta (Via Ferdinando Palasciano - 81100 Caserta (CE)). Most of the isolates belonged to patients from European countries (n = 98) and African countries (n = 44), only six and five subjects came from Asia and South America respectively (for six cases no information was available), respectively. MTB isolates were isolated from both pulmonary (n = 140) and extrapulmonary (n = 19) sites. All these data are summarised in Tables 1–4.

**Table 1:** Origin and phenotypic antibiotic resistance profile of the *M. tuberculosis* strains isolated at AORN S.G. Moscati (N.R., not received; N.T., not tested).

Hospital	Sample ID.	Patient’s provenance	Biological material	Phenotypic antibiotic resistance profile					
				RFP	INH	ETH	STR	PRZ	EMB
AORN S.G. Moscati	2018_AM_1_S77	Mali	Abscess	S	S	S	S	S	N.T.
	2018_AM_2_S78	Italy	Bronchoaspirate	S	S	S	S	S	N.T.
	2018_AM_3_S79	Somalia	Bronchoalveolar lavage	S	S	S	R	S	N.T.
	2018_AM_5_S81	Italy	Bronchoalveolar lavage	S	S	S	S	S	N.T.
	2018_AM_6_S82	Italy	Bronchoaspirate	S	S	S	S	S	N.T.
	2018_AM_7_S64	N.R.	Bronchoaspirate	R	S	S	S	S	N.T.
	2019_AM_9_S84	Italy	Bronchoaspirate	S	S	S	S	S	N.T.
	2019_AM_10_S85	Gambia	Peritoneal fluid	S	S	S	S	N.T.	N.T.
	2019_AM_11_S86	Romania	Sputum	S	S	S	S	S	N.T.
	2019_AM_12_S87	Italy	Bronchoaspirate	S	S	S	S	S	N.T.
	2019_AM_13_S88	Guinea-Bissau	Pus	S	S	S	S	S	N.T.
	2019_AM_14_S89	Italy	Lymph node fluid	S	S	S	S	S	N.T.
	2019_AM_15_S90	Somalia	Sputum	S	R	R	S	S	N.T.
	2019_AM_16_S91	N.R.	Sputum	S	R	S	R	S	N.T.
	2020_AM_17_S92	N.R.	Sputum	S	S	S	S	S	N.T.
	2020_AM_18_S93	Italy	Sputum	S	S	S	S	S	N.T.
	2020_AM_19_S94	Italy	Bronchoalveolar lavage	S	S	S	S	S	N.T.
	2021_AM_20_S95	N.P.	Bronchoaspirate	S	S	S	S	S	N.T.
	2020_AM_21_S65	Italy	Bronchoalveolar lavage	S	S	S	S	S	N.T.
	2021_AM_22_S66	Somalia	Pus	S	S	S	S	S	N.T.
	2021_AM_24_S67	India	Bronchoaspirate	S	S	S	S	S	N.T.
	2021_AM_25_S68	Gambia	Pleural fluid	S	S	S	S	S	N.T.
	2021_AM_27_S96	Gambia	Bronchoaspirate	S	S	S	S	S	N.T.

**Table 2:** Origin and phenotypic antibiotic resistance profile of the *M. tuberculosis* strains isolated at Ascalesi hospital (N.R., not received; N.T., not tested).

Hospital	Sample ID.	Patient’s provenance	Biological material	Phenotypic antibiotic resistance profile					
				RFP	INH	ETH	STR	PRZ	EMB
Ascalesi hospital	2019_AS_1_S20	Ukraine	Sputum	S	S	S	S	S	N.T.
	2019_AS_2_S63	Somalia	Lymph node fluid	S	S	S	S	S	N.T.
	2019_AS_3_S21	Italy	Sputum	S	S	S	S	S	N.T.
	2019_AS_4_S22	Bulgaria	Sputum	S	S	S	S	S	N.T.
	2019_AS_5_S23	China	Sputum	S	S	S	S	S	N.T.
	2019_AS_6_S24	Bangladesh	Cerebrospinal fluid	S	S	S	S	S	N.T.
	2019_AS_7_S25	Bulgaria	Sputum	S	S	S	S	S	N.T.
	2019_AS_8_S26	Perù	Sputum	S	S	S	S	S	N.T.
	2019_AS_9_S27	Romania	Sputum	S	S	S	S	S	N.T.
	2019_AS_10_S28	Senegal	Sputum	S	S	S	S	S	N.T.
	2019_AS_11_S29	Russia	Sputum	S	S	S	S	S	N.T.
	2019_AS_12_S30	Senegal	Ascitic fluid	S	S	S	S	S	N.T.
	2019_AS_13_S31	Bulgaria	Ascitic fluid	S	S	S	S	S	N.T.
	2019_AS_14_S32	Italy	Sputum	S	S	S	S	S	N.T.
	2019_AS_15_S33	Ukraine	Sputum	S	S	S	S	S	N.T.
	2019_AS_16_S34	Italy	Bronchoaspirate	S	S	S	S	S	N.T.
	2019_AS_17_S35	Perù	Sputum	S	S	S	S	S	N.T.
	2019_AS_19_S36	Senegal	Sputum	S	S	S	S	S	N.T.
	2020_AS_27_S1	Romania	Sputum	S	S	S	S	S	N.T.
	2020_AS_28_S2	Bulgaria	Pleural fluid	S	S	S	S	S	N.T.
	2020_AS_29_S33	East-Europe	Sputum	S	S	S	S	S	N.T.
	2020_AS_30_S47	Ukraine	Sputum	S	S	S	S	S	N.T.
	2020_AS_31_S34	Italy	Sputum	S	S	S	S	S	N.T.
	2020_AS_32_S35	Perù	Pleural fluid	S	S	S	S	S	N.T.
	2020_AS_33_S36	Italy	Ascitic fluid	S	S	S	S	S	N.T.
	2020_AS_34_S48	Romania	Sputum	S	S	S	S	S	N.T.
	2021_AS_35_S37	Bulgaria	Sputum	S	S	S	S	S	N.T.
	2021_AS_36_S38	Senegal	Sputum	S	S	S	S	R	N.T.
	2021_AS_37_S39	Romania	Sputum	S	S	S	S	S	N.T.
	2021_AS_38_S83	Italy	Lymph node biopsy	S	S	S	S	R	N.T.
	2021_AS_39_S49	Russia	Sputum	S	S	S	S	S	N.T.
	2021_AS_40_S3	East-Europe	Sputum	S	S	R	S	S	N.T.
	2021_AS_41_S4	Russia	Sputum	R	R	S	R	S	N.T.
	2021_AS_42_S50	Russia	Sputum	S	S	S	S	R	N.T.
	2021_AS_43_S51	Bulgaria	Sputum	S	S	S	S	S	N.T.
	2021_AS_44_S5	Ukraine	Sputum	S	R	S	S	S	N.T.
	2021_AS_45_S40	Senegal	Sputum	S	S	S	S	S	N.T.
	2021_AS_46_S52	Romania	Sputum	S	S	S	S	S	N.T.
	2021_AS_47_S56	Perù	Sputum	S	S	S	S	S	N.T.
	2021_AS_48_S41	East-Europe	Sputum	S	S	S	S	S	N.T.
	2021_AS_49_S6	Ukraine	Sputum	S	S	S	S	S	N.T.

**Table 3:** Origin and phenotypic antibiotic resistance profile of the *M. tuberculosis* strains isolated at AO Sant’Anna and San Sebastiano (N.R., not received; N.T., not tested).

Hospital	Sample ID.	Patient’s provenance	Biological material	Phenotypic antibiotic resistance profile					
				RFP	INH	ETH	STR	PRZ	EMB
AO Sant’Anna e San Sebastiano	2020_SS_1_S1	Romania	Sputum	S	S	S	S	N.T.	N.T.
	2020_SS_2_S2	Italy	Sputum	S	S	S	S	N.T.	N.T.
	2019_SS_3_S3	Italy	Sputum	S	S	S	S	N.T.	N.T.
	2019_SS_4_S4	Italy	Sputum	S	S	S	S	N.T.	N.T.
	2019_SS_6_S6	Italy	Sputum	S	S	S	S	N.T.	N.T.
	2019_SS_7_S7	Italy	Sputum	S	S	S	S	N.T.	N.T.
	2020_SS_9_S62	Marocco	N.R.	S	S	S	S	N.T.	N.T.
	2019_SS_16_S14	Italy	Bronchoaspirate	S	S	S	S	N.T.	N.T.
	2019_SS_17-1_S7	India	Sputum	S	S	S	S	N.T.	N.T.
	2019_SS_18-1_S8	Nigeria	Sputum	S	S	S	S	N.T.	N.T.
	2019_SS_19-1_S42	Marocco	Sputum	S	S	S	S	N.T.	N.T.
	2019_SS_20-1_S43	Romania	Sputum	S	S	S	S	N.T.	N.T.
	2019_SS_21-1_S9	Ukraine	Sputum	S	S	S	S	N.T.	N.T.
	2019_SS_22-1_S10	China	Sputum	S	S	S	S	N.T.	N.T.
	2019_SS_23-1_S11	Marocco	Bronchoaspirate	R	R	S	R	N.T.	N.T.
	2019_SS_24-1_S57	Marocco	Bronchoaspirate	N.T.	S	S	S	N.T.	N.T.
	2019_SS_25-1_S53	Ukraine	Sputum	N.T.	S	S	S	N.T.	N.T.
	2019_SS_26-1_S44	Nigeria	Bronchoaspirate	S	S	S	S	N.T.	N.T.
	2019_SS_27-1_S12	Marocco	Sputum	S	S	S	S	N.T.	N.T.
	2019_SS_28-1_S13	Nigeria	Sputum	S	S	S	S	N.T.	N.T.
	2021_SS_29-1_S14	Marocco	Sputum	S	S	S	S	N.T.	N.T.
	2019_SS_30_S15	Marocco	Sputum	S	S	S	S	N.T.	N.T.
	2019_SS_31_S16	Italy	Sputum	S	S	S	S	N.T.	N.T.
	2019_SS_32-1_S16	Italy	Sputum	S	S	S	S	N.T.	N.T.
	2020_SS_33-1_S17	Italy	Bronchoaspirate	S	S	S	S	N.T.	N.T.
	2021_SS_35_S17	Italy	Sputum	S	S	S	S	N.T.	N.T.
	2021_SS_37-1_S19	Marocco	Sputum	S	S	S	S	N.T.	N.T.
	2021_SS_39_S19	Marocco	Bronchoaspirate	S	S	S	S	N.T.	N.T.
	2021_SS_39-1_S45	N.R.	Bronchoaspirate	S	S	S	S	N.T.	N.T.

**Table 4:** Origin and phenotypic antibiotic resistance profile of M. tuberculosis strains isolated at Cotugno hospital (N.R., not received; N.T., not tested).

Hospital	Sample ID.	Patient’s provenance	Biological material	Phenotypic antibiotic resistance profile					
				RFP	INH	ETH	STR	PRZ	EMB
Cotugno hospital	2019_CO_1_S37	Tunisia	Sputum	S	S	N.T.	S	N.T.	S
	2019_CO_2_S38	Ukraine	Sputum	S	S	N.T.	S	N.T.	S
	2019_CO_3_S39	Italy	Sputum	S	S	N.T.	S	N.T.	S
	2019_CO_5_S40	Ukraine	Sputum	S	S	N.T.	S	N.T.	S
	2019_CO_6_S41	Italy	Cerebrospinal fluid	S	S	N.T.	S	N.T.	S
	2019_CO_8_S42	Nigeria	Sputum	S	S	N.T.	S	N.T.	S
	2019_CO_9_S43	Marocco	Sputum	S	R	N.T.	S	N.T.	S
	2019_CO_10_S44	Tunisia	Sputum	S	S	N.T.	S	N.T.	S
	2019_CO_11_S70	Ghana	Sputum	S	S	N.T.	S	N.T.	S
	2019_CO_12_S45	Marocco	Sputum	S	S	N.T.	S	N.T.	S
	2019_CO_13_S46	Albania	Sputum	S	S	N.T.	S	N.T.	S
	2019_CO_14_S47	Italy	Sputum	S	S	N.T.	S	N.T.	S
	2019_CO_15_S48	Italy	Bronchoaspirate	S	S	N.T.	S	N.T.	S
	2019_CO_16_S49	China	Sputum	S	S	N.T.	S	N.T.	S
	2019_CO_17_S71	Romania	Sputum	S	R	N.T.	S	N.T.	S
	2019_CO_18_S50	Romania	Sputum	S	S	N.T.	R	N.T.	S
	2019_CO_19_S58	Gambia	Skin	S	S	N.T.	S	N.T.	S
	2019_CO_20_S51	Italy	Bronchoaspirate	S	R	N.T.	S	N.T.	S
	2019_CO_21_S46	Romania	Sputum	S	S	N.T.	S	N.T.	S
	2019_CO_22_S59	Marocco	Sputum	S	R	N.T.	S	N.T.	S
	2019_CO_23_S72	Ciad	Sputum	S	S	N.T.	S	N.T.	S
	2019_CO_24_S52	Romania	Sputum	S	S	N.T.	S	N.T.	S
	2019_CO_25-1_S53	Romania	Sputum	S	S	N.T.	S	N.T.	S
	2019_CO_26_S74	Senegal	Sputum	S	S	N.T.	S	N.T.	S
	2019_CO_27_S75	Nigeria	Sputum	S	S	N.T.	S	N.T.	S
	2019_CO_28_S76	N.R.	Sputum	S	S	N.T.	S	N.T.	S
	2019_CO_29_S53	Italy	Sputum	S	S	N.T.	S	N.T.	S
	2019_CO_30_S54	Italy	Sputum	S	S	N.T.	S	N.T.	S
	2019_CO_31_S55	Italy	Sputum	S	S	N.T.	S	N.T.	S
	2019_CO_32_S56	Italy	Peritoneal fluid	S	S	N.T.	S	N.T.	S
	2019_CO_33_S77	Italy	Cerebrospinal fluid	S	S	N.T.	S	N.T.	S
	2019_CO_34_S57	Italy	Bronchoaspirate	S	R	N.T.	S	N.T.	S
	2019_CO_35_S78	Senegal	Sputum	S	S	N.T.	S	N.T.	S
	2019_CO_36_S79	N.R.	Sputum	S	S	N.T.	S	N.T.	S
	2019_CO_37_S80	Somalia	Sputum	S	S	N.T.	S	N.T.	S
	2019_CO_38_S58	Romania	Sputum	S	S	N.T.	S	N.T.	S
	2019_CO_39_S59	Italy	Sputum	S	S	N.T.	S	N.T.	S
	2019_CO_40_S60	Italy	Sputum	S	S	N.T.	S	N.T.	S
	2019_CO_41_S81	N.R.	Sputum	S	S	N.T.	S	N.T.	S
	2019_CO_42_S61	Italy	Sputum	S	S	N.T.	R	N.T.	S
	2019_CO_43_S62	Italy	Sputum	S	S	N.T.	S	N.T.	S
	2019_CO_44_S63	Italy	Bronchoaspirate	S	S	N.T.	S	N.T.	S
	2019_CO_45_S64	Poland	Sputum	S	S	N.T.	S	N.T.	S
	2019_CO_46_S65	Marocco	Sputum	S	S	N.T.	S	N.T.	S
	2019_CO_47_S66	Italy	Sputum	S	S	N.T.	S	N.T.	S
	2019_CO_48_S67	Italy	Sputum	S	S	N.T.	S	N.T.	S
	2019_CO_49_S68	Italy	Peritoneal fluid	S	S	N.T.	S	N.T.	S
	2019_CO_50_S54	Romania	Sputum	S	S	N.T.	S	N.T.	S
	2019_CO_51_S60	Poland	Sputum	S	S	N.T.	S	N.T.	S
	2019_CO_52_S69	Italy	Bronchoaspirate	S	S	N.T.	S	N.T.	S
	2019_CO_53_S70	Ciad	Sputum	S	S	N.T.	S	N.T.	S
	2019_CO_54_S71	Burkina	Sputum	S	S	N.T.	S	N.T.	S
	2019_CO_55_S72	Somalia	Sputum	S	S	N.T.	S	N.T.	S
	2019_CO_56_S73	Italy	Sputum	S	S	N.T.	S	N.T.	S
	2019_CO_57_S74	Italy	Sputum	S	S	N.T.	S	N.T.	S
	2019_CO_58_S75	Italy	Sputum	S	S	N.T.	S	N.T.	S
	2019_CO_59_S61	Italy	Sputum	S	S	N.T.	S	N.T.	S
	2019_CO_60_S82	Mali	Sputum	S	S	N.T.	S	N.T.	S
	2019_CO_61_S76	Romania	Sputum	S	S	N.T.	S	N.T.	S
	2019_CO_63-1_S20	Italy	Sputum	S	S	N.T.	S	N.T.	S
	2019_CO_64-1_S21	Ukraine	Sputum	S	S	N.T.	S	N.T.	S
	2019_CO_65-1_S26	Italy	Sputum	S	S	N.T.	S	N.T.	S
	2019_CO_66-1_S22	Romania	Sputum	S	S	N.T.	S	N.T.	S
	2019_CO_68-1_S23	Italy	Sputum	S	S	N.T.	S	N.T.	S
	2019_CO_69-1_S27	Romania	Sputum	S	S	N.T.	S	N.T.	S
	2019_CO_70-1_S24	Italy	Sputum	S	S	N.T.	S	N.T.	S

### Ethical considerations

The study protocol was subjected to review by the ethics committee of the participating hospitals. Following a thorough assessment, the committee determined that the samples under investigation were not human, obviating the need for ethical approval for the study. Furthermore, the bacterial strains exploited for this research were subjected to anonymization through the application of a distinctive identification code, encrypted to safeguard the privacy of the subjects involved. Consequently, obtaining informed consent from patients was deemed unnecessary.

### MTB isolates cultivation and antibiogram

The bacterial clinical isolates examined in this study were obtained from patients as part of routine diagnostic requests conducted by collaborating hospitals. Biological samples were processed using the standard N-acetyl-1-cysteine-sodium hydroxide (NALC-NaOH) method for digestion, decontamination, and concentration of bacterial load. The pellet was resuspended in approximately 2 mL of phosphate buffer (pH 7) and mixed thoroughly. The suspension was used for microscopic analysis and for setting up bacterial culture. A volume of 250 and 500 μl of the suspension were inoculated in BACTEC MGIT 960 (Becton Dickinson, Franklin Lakes, NJ, USA) and on solid culture media Löwenstein-Jensen (LJ). Identification of mycobacterial species was performed by IS6110-based PCR. Five first-line drugs including streptomycin (STM, 1.0 μg/mL), isoniazid (INH, 0.1 μg/mL), rifampicin (RIF, 1.0 μg/mL), Pyrazinamide (PRZ, 100 μg/mL) and ethambutol (EMB, 5 μg/mL) were tested using the Mycobacterium Growth Indicator Tube 960 (MGIT 960) system^20^. After identification and susceptibility determination, bacterial stocks were prepared, catalogued, and stored at −80 °C in accordance with standard hospital practices. In relation to the study, MTB isolates were re-inoculated in BACTEC MGIT 960 culture tube. Bacterial culture was centrifuged, and genomic DNA extraction was applied to the pellet.

### Genomic DNA extraction and whole-genome sequencing

Genomic DNA extractions were performed at the hospitals where the strains were isolated. Genomic DNA was obtained from clinical strains using two column-based DNA extraction methods (QIAamp DNA minikit, Qiagen, Germany), according to the instructions. Total DNA concentration was determined by using Quant-IT DNA Assay Kit and a Qubit Fluorometer (Life Technologies, Monza, Italy) and its purity was determined by using NanoDrop spectrophotometer ND-2000 (Thermo Fischer Scientific) through the evaluation of the absorbance ratio A260/A280 and A230/A280. Library preparation and sequencing processes were carried out at Laboratory of Molecular Medicine and Genomics, a research lab of the University of Salerno (Italy). Indexed libraries were prepared starting from 60 ng of each DNA sample according to Illumina DNA Prep sample preparation kits (Illumina Inc., San Diego, CA, USA). Final library concentrations and size were assessed with Quant-IT DNA Assay Kit and Agilent 4200 Tapestation System (Agilent Technologies, Milan, Italy), respectively. Then, 159 libraries were equimolarly pooled, diluted to a final concentration of 1.3 pMol and sequenced in paired-end, 300 bp, on the Illumina NexSeq. 500 platform (Illumina, San Diego, CA).

### Genomic analysis

The sequenced reads were quality checked with FastQC v0.11.3^21^. The low-quality and adapter-related fragments were removed using cutadapt v 1.18 with the following parameters: -m = 20 (minimum read length); -q = 20 (minimum read quality)^22^. The high-quality reads were then imported into TB-profiler^23^ with–min_depth option set to 50, to retain only mutation supported by at least 50 reads. TB-profiler (v4.2.0) analysis allowed lineages assignment and antimicrobial susceptibility prediction (drug resistance) working with MTB H37Rv reference genome (GenBank accession: GCA_000195955.2) and resistance is predicted using the curated database provided with TB-profiler software. This database has been tested using over 17,000 samples with genotypic and phenotypic MTBC data. The phylogenetic trees were inferred based on the whole genome single nucleotide polymorphism, as proposed by Senghore *et al*. using TB-profiler intermediate alignment files^24^. In detail,.bam files were processed with freebayes v.1.3.5 to call variants^25^ using the following parameters: -p 1,–min-coverage 5, -q 20. Then, variant calling files (.vcf) were processed with snippy v3.1^26^ to filter out non-significant mutation using the snippy-vcf_filter function, with–minfrac and–minqual parameters set to 0.1 and 20 respectively. The bcftools software (version 1.12)^27^ has been used to generate consensus sequences for each isolate, which have been given in input to JolyTree (v.1.1b.191021ac)^28^ to compute a fast distance-based phylogenetic inference from unaligned genome sequences. Finally, JolyTree output files (.newick) have been used as input to iTol online software^29^ for tree construction and visualisation.

## Data Records

The DNA sequencing have been deposited in the EBI ArrayExpress database (http://www.ebi.ac.uk/arrayexpress) with accession number E-MTAB-13058 that contains the raw data related to complete WGS from all samples analyzed in the study^30^.

## Technical Validation

The reliability of the dataset yielded from this study was evaluated based on multiple aspects. To verify the quality and robustness of the data presented, MTB isolates were grown in a proper selective medium, identified and characterized for their phenotypic antibiotic resistance profile, according to standard procedures (Tables 1–4). In this context, the use of the MGIT 960 system in conjunction with the antibiogram assays resulted in a valid and rapid method to reveal the presence of the mycobacterium and to confirm MTB-infected patients, highlighting the diverse distribution of both sensitive and drug-resistant isolates within each hospital participating in the study. Supplementary File 1 and Fig. 2 provide an evaluation of the quality of the WGS data generated, including the mapping percentage on the MTB reference genome, lineage distribution and resistance profiles.

Starting from the extracted genomic DNA, whole genome sequencing was performed. The sequencing of 159 MTB isolates produced in total 424,760,352 reads (range 1,392,456 −5,035,024 reads), corresponding to an average of 2,671,448.75 reads per sample. After low-quality reads filtering and adapter removal, 424,511,352 reads (2,669,882.72 reads per sample, range 1,391,848 - 5,032,296 reads) remained for downstream analysis. Our results showed an overall high percentage of mapping, about 98.24% of high-quality reads per sample, in fact, aligned on the established MTB reference genome (H37Rv), along with a median coverage value of 66.9 per sample. The *in silico* analysis of the 159 isolates detected eight different MTB lineages: lineage1 (n = 4), lineage2 (n = 5), lineage3 (n = 12), lineage4 (n = 129), lineage5 (n = 1), lineage6 (n = 5) and lineage2_lineage4 (n = 2) and lineage9 (n = 1). Among all hospitals enrolled, the dominant lineage was Euro-American 4 (n = 129, 81.13%), with 32 identified at Ascalesi Hospital, 61 at Cotugno Hospital, 15 at Moscati Hospital and 21 at Sant’Anna e San Hospital Sebastian. All lineage assignments are summarized in Fig. 2b. In the context of antibiotic resistance, approximately ~12.6% of samples (20/159 samples) were nonsusceptible while 139 MTB isolates were classified as antibiotic susceptible.

In detail, eight, four and one isolate were reported as HR-TB, MDR-TB and Pre-XDR-TB respectively, while for seven samples resistance to only one drug was found, as reported in Fig. 2a. The antibiogram results were in agreement with the data obtained from drug resistance analysis by WGS, showing a high percentage of correspondence between in silico prediction and antibiotic tests. In fact, approximately 93.3% of the drugs tested (653/700 tests) showed the same trend in the WGS prediction analysis. Despite the limited prevalence of MDR-MTB isolated in relation to the overall size of the sample, the integration of this set of data with comparable ones, accompanied by rigorous data validation procedures, can basically improve the statistical power of the analysis. In addition, this integration could significantly contribute to the advancement of new therapies and diagnostic tools in the ongoing battle against TB.

### Supplementary information

Supplementary file 1

## Data Availability

Different tools have been employed for data analysis, and the following sections describe their versions, settings, and parameters: • FastQC (v0.11.3) with default parameters; • cutadapt (v1.18) with the following parameters: -m 20, -q 20; • TB-profiler (v4.2.0) with the following parameter: --min_depth 50; • freebayes (v1.3.5) with the following parameters: -p 1, --min-coverage 5, -q 20; • snippy (v3.1) with the following parameters: snippy-vcf_filter, --minfrac 0.1, --minqual 20; • bcftools (v1.12) with default parameters; • JolyTree (v1.1b.191021ac) with default parameters; • iTol online software.

## References

[1] Khan FY (2019). Review of literature on disseminated tuberculosis with emphasis on the focused diagnostic workup. J Family Community Med..

[2] Viney K, Islam T, Hoa NB, Morishita F, Lönnroth K (2019). The Financial Burden of Tuberculosis for Patients in the Western-Pacific Region. Trop Med Infect Dis..

[3] European Centre for Disease Prevention and Control (ECDC). *Survaillance Atlas of Infectious Diseases*. https://atlas.ecdc.europa.eu/public/index.aspx (2022).

[4] Zhang H, Liu M, Fan W, Sun S, Fan X (2022). The impact of Mycobacterium tuberculosis complex in the environment on one health approach. Front Public Health..

[5] Borrell S (2019). Reference set of Mycobacterium tuberculosis clinical strains: A tool for research and product development. PLoS One..

[6] Silva ML (2022). Tuberculosis caused by Mycobacterium africanum: Knowns and unknowns. PLoS Pathog..

[7] O’Neill MB (2019). S. Lineage specific histories of Mycobacterium tuberculosis dispersal in Africa and Eurasia. Mol Ecol..

[8] Stucki D (2016). Mycobacterium tuberculosis lineage 4 comprises globally distributed and geographically restricted sublineages. Nat Genet..

[9] Diarra B (2018). Mycobacterium africanum (Lineage 6) shows slower sputum smear conversion on tuberculosis treatment than Mycobacterium tuberculosis (Lineage 4) in Bamako, Mali. PLoS One..

[10] Coscolla M (2021). Phylogenomics of Mycobacterium africanum reveals a new lineage and a complex evolutionary history. Microb Genom..

[11] Liebenberg, D., Gordhan, B.G., Kana, B.D. Drug resistant tuberculosis: Implications for transmission, diagnosis, and disease management. *Front Cell Infect Microbiol*. **12**, 943545. 10.3389/fcimb.2022.943545 PMC9538507 36211964

[12] Yeboah-Manu D, de Jong BC, Gehre F (2017). The Biology and Epidemiology of Mycobacterium africanum. Adv Exp Med Biol..

[13] Tientcheu LD (2014). Differences in T-cell responses between Mycobacterium tuberculosis and Mycobacterium africanum-infected patients. Eur J Immunol..

[14] de Jong BC, Antonio M, Gagneux S (2014). Mycobacterium africanum–review of an important cause of human tuberculosis in West Africa. PLoS Negl Trop Dis..

[15] Yimer SA (2020). Lineage-Specific Proteomic Signatures in the Mycobacterium tuberculosis Complex Reveal Differential Abundance of Proteins Involved in Virulence, DNA Repair, CRISPR-Cas, Bioenergetics and Lipid Metabolism. Front Microbiol..

[16] Shanmugam SK (2022). Mycobacterium tuberculosis Lineages Associated with Mutations and Drug Resistance in Isolates from India. Microbiol Spectr..

[17] World Health Organization (WHO). *Global Tuberculosis Programme*. https://www.who.int/teams/global-tuberculosis-programme (2023).

[18] Karuniawati A (2022). Performance of Xpert MTB/RIF and sputum microscopy compared to sputum culture for diagnosis of tuberculosis in seven hospitals in Indonesia. Front Med (Lausanne)..

[19] Madrazo-Moya CF (2019). Whole genomic sequencing as a tool for diagnosis of drug and multidrug-resistance tuberculosis in an endemic region in Mexico. PLoS One..

[20] Gill CM, Dolan L, Piggott LM, McLaughlin AM (2022). New developments in tuberculosis diagnosis and treatment. Breathe (Sheff)..

[21] Andrews, S. *FastQC: A Quality Control Tool for High Throughput Sequence Data*. http://www.bioinformatics.babraham.ac.uk/projects/fastqc/ (2010).

[22] Martin M (2011). Cutadapt removes adapter sequences from high-throughput sequencing reads. EMBnet J..

[23] Phelan JE (2019). Integrating informatics tools and portable sequencing technology for rapid detection of resistance to anti-tuberculous drugs. Genome Med..

[24] Senghore M (2020). Evolution of Mycobacterium tuberculosis complex lineages and their role in an emerging threat of multidrug resistant tuberculosis in Bamako, Mali. Sci Rep..

[25] Garrison, E., Marth, G. Haplotype-based variant detection from short-read sequencing. Published online (2012).

[26] Seemann T snippy. *Fast bacterial variant calling from NGS reads* https://github.com/tseemann/snippy (2015)

[27] Li Y, Xiao P, Wang Y, Hao Y (2020). Mechanisms and Control Measures of Mature Biofilm Resistance to Antimicrobial Agents in the Clinical Context. ACS Omega..

[28] Criscuolo A (2020). A fast alignment-free bioinformatics procedure to infer accurate distance-based phylogenetic trees from genome assemblies. RIO..

[29] Letunic I, Bork P (2021). Interactive Tree Of Life (iTOL) v5: an online tool for phylogenetic tree display and annotation. Nucleic Acids Res..

[30] Ferravante C, Giurato G, Weisz A, Nassa G, Franci G (2023). ArrayExpress.

